# A rare case of anaphylaxis to Indian jujube (*Ziziphus Mauritiana Lam*)

**DOI:** 10.1186/s13223-020-00444-y

**Published:** 2020-06-17

**Authors:** Babak Aberumand, Rozita Borici-Mazi

**Affiliations:** 1 Department of Medicine, Queen’s University, Kingston, ON Canada; 2 Division of Allergy & Immunology, Department of Medicine, Queen’s University, Hotel Dieu Hospital, 166 Brock Street, Kingston, ON K7L 5G2 Canada

**Keywords:** Indian jujube, Anaphylaxis, *Ziziphus Mauritiana Lam*, Food allergy

## Abstract

**Background:**

Indian jujube (*Ziziphus Mauritiana Lam*) is a sweet fruit from a tree native to tropical and subtropical regions of Asia and India. A few case reports have implicated Indian jujube to cause latex-fruit syndrome. We present the first case of an anaphylactic reaction to this fruit in a patient with no latex allergy.

**Case presentation:**

A 55-year-old male was referred to the Outpatient Allergy Clinic at Queen’s University for evaluation of anaphylaxis caused by ingestion of Indian jujube. He presented to the Emergency Department (ED) with scalp pruritus, dyspnea and generalized urticaria, which occurred two hours after he had consumed a homemade candied fruit cocktail consisting of Indian jujube, water, Thai and Indian sweetener. In the ED, he was treated with epinephrine, intravenous diphenhydramine and steroids. He did not have any previous history of environmental or food allergies but had consumed this fruit frequently since childhood. In clinic, he underwent skin-prick testing with a saline slurry of candied jujube, which resulted in a positive wheal and flare response with appropriate controls. On subsequent visit, skin-prick tests were performed with saline slurries of the Thai and Indian sweetener used to make the cocktail. Both tests were negative when applied to a healthy volunteer. Skin-prick testing to latex allergen and latex specific IgE were both negative. He was diagnosed with an IgE-mediated anaphylactic reaction to the Indian jujube fruit. He was advised to avoid consumption of Indian jujubes and carry an epinephrine autoinjector.

**Conclusions:**

Anaphylaxis secondary to Indian jujube ingestion is an extremely rare phenomenon in patients without a latex allergy. A possible allergy to Indian jujube should be taken into consideration when working up anaphylaxis, especially in patient of Asian and Indian descent who have ceased regular consumption of the fruit.

## Background

Indian jujube (*Ziziphus Mauritiana Lam*), also known as Ber, is a tree of the family *Rhamnaceae* that bears a sweet fruit. It is native to tropical and subtropical regions of Asia that include Southern China, India and Malaysia [1–3]. A few case reports have described a latex-fruit syndrome where there is a cross-reaction between the allergenic components of latex and the Indian jujube [1–5]. We present the first case of an anaphylactic reaction to this fruit in a patient without a latex allergy.

## Case presentation

A 55-year-old man of Bangladesh descent was referred to the Outpatient Allergy Clinic at Queen’s University for evaluation of anaphylaxis caused by ingestion of Indian jujube. He presented to the Emergency Department (ED) with symptoms of scalp pruritus, dyspnea and generalized urticaria. This occurred 2 h after he had consumed a homemade candied fruit cocktail consisting of Indian jujube, water, Thai and Indian sweetener (made by locally grown sugar cane from Thailand and India). In ED, anaphylaxis was diagnosed and symptom resolution was obtained with epinephrine, intravenous diphenhydramine and steroids. The patient did not have any known history of environmental or food allergies. He grew up with a *Z. Mauritiana Lam* tree in his backyard and regularly consumed Indian jujubes throughout his life, since childhood. However, when he moved to Canada, its consumption considerably decreased and last time he ingested Indian jujube was 5–6 months prior to this event. He reported no other episodes of anaphylaxis over lifetime. He had a medical history comprising of hypertension, type 2 diabetes mellitus and dyslipidemia. His medications’ list included hydrochlorothiazide 25 mg once daily, ramipril 10 mg once daily, metformin 500 mg twice daily, and simvastatin 25 mg once daily.

**Fig. 1:**
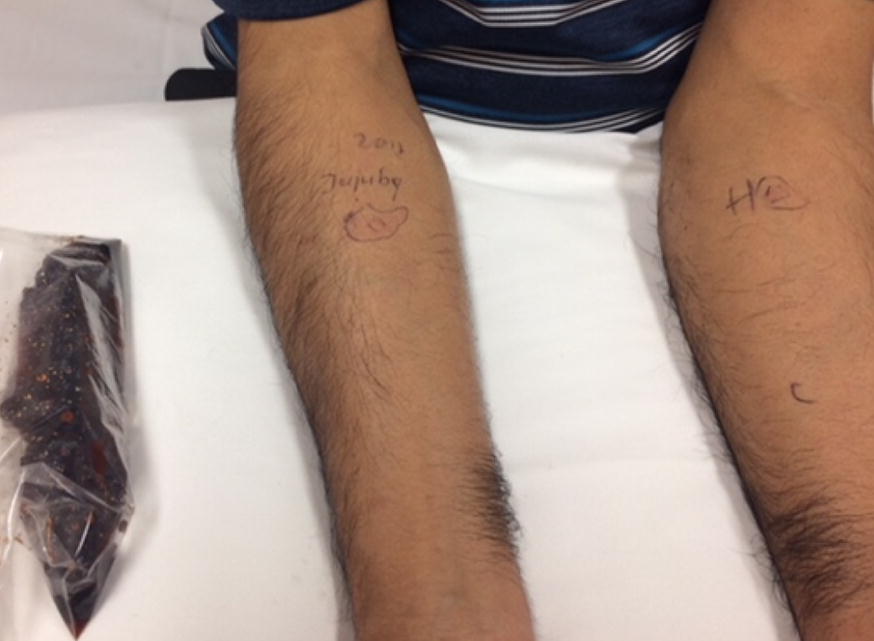
Wheal and flare response to SPT with slurry of candid jujube. H and C indicate histamine and saline control, respectively

**Fig. 2:**
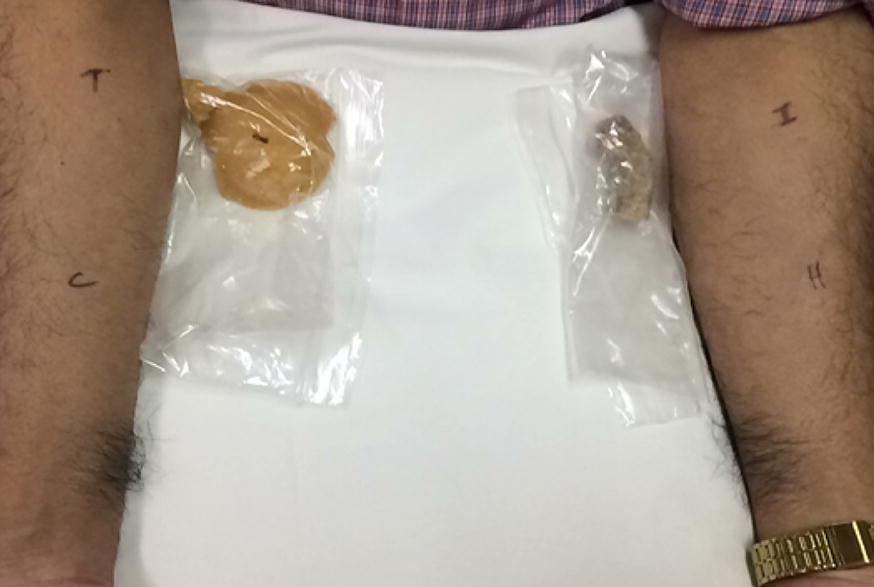
Wheal and flare response to skin-prick test to saline slurry of Thai (T) and Indian (I) sweeteners. H and C indicate histamine and saline control, respectively

**Fig. 3:**
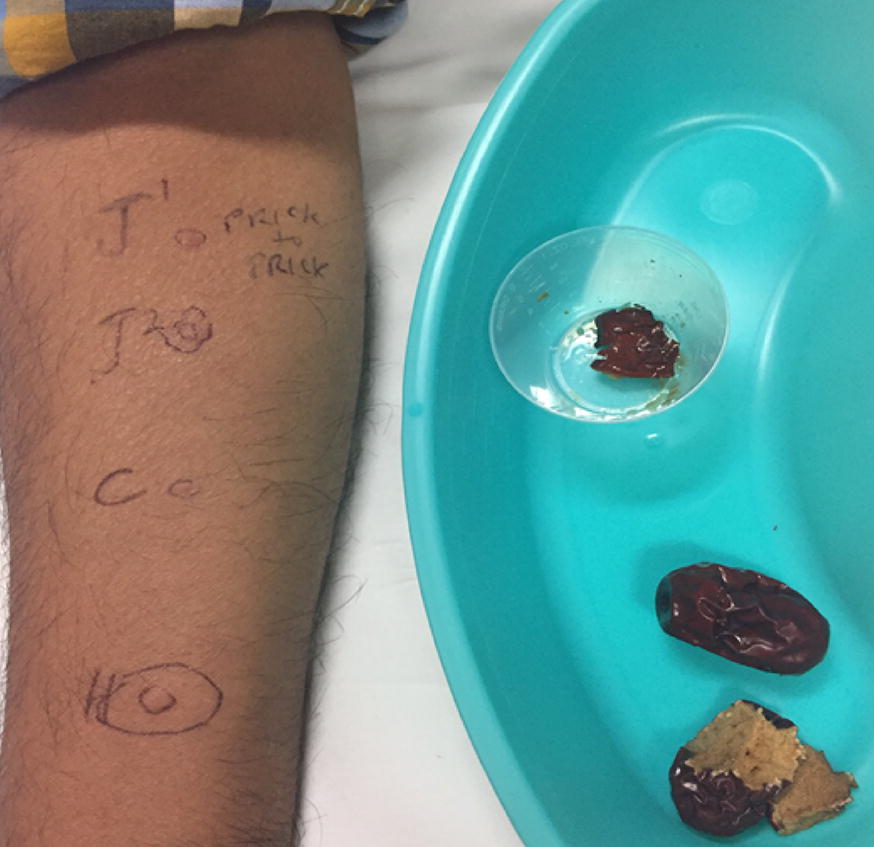
Wheal and flare response to prick- to- prick test (J1) and SPT through dried jujube soaked in normal saline, applied on skin (J2). H and C indicate histamine and saline control, respectively

In clinic, we performed skin-prick testing with various formulations of Indian jujube, environmental and latex allergens. The skin -prick testing (SPT) was considered positive when the longest diameter of the wheal response was 3 mm greater than the saline control. SPT to a saline slurry (1/20 weight/volume) of candied jujube resulted in a wheal and flare response (W&F) with longest diameters of 5 mm and 25 mm (5 × 25 mm), respectively, with appropriate controls (Fig. 1). Subsequently, SPT were performed with saline slurries (1/10 weight/volume) of the Thai and Indian sweeteners used to make the cocktail, which were negative with appropriate controls (Fig. 2). On a further visit, he underwent prick-to-prick testing with dried Indian jujube soaked in normal saline, which resulted in a W&F response measuring 4 × 5 mm. Subsequently, SPT through dried jujube soaked in normal saline and applied on the forearm caused a W&F reaction measuring 5 × 15 mm; W&F response to histamine was 6 × 30 mm and to saline control was 1 × 4 mm. (Figure 3). Specific IgE to Indian jujube was not available. All tests were applied to a healthy volunteer and no false positives were noted. Latex allergy was excluded based on tolerance of latex products, as well as negative SPT and serum latex IgE level (< 0.10 kU/L). A SPT to a panel of common environmental allergens demonstrated a positive W&F response to dust mite, bird feathers and showed no tree, grass or ragweed pollen sensitivity. He was diagnosed with an IgE-mediated anaphylactic reaction to Indian jujube, without a latex-fruit syndrome. He was advised to avoid consumption of Indian jujubes and carry an epinephrine autoinjector.

## Discussion and conclusion

Food induced anaphylaxis is a severe and life-threatening allergic reaction that can be caused by the ingestion of a food to which a patient is allergic. The IgE-mediated mechanism involves the release of inflammatory mediators from mast cells and basophils after cross linkage of food allergen with allergen-specific IgE antibodies bound to these cells [6]. We report a case of IgE-mediated anaphylaxis after consumption of Indian jujube. The diagnosis was based on clinical temporal correlation of systemic symptoms suggestive of anaphylaxis upon consumption of this fruit. His symptoms responded to administration of epinephrine and antihistamines, although ED physician prescribed adjunctive therapy with steroids, which was not necessarily indicated. A tryptase level at the time of the presentation to ED would have been helpful, but it was not ordered. The diagnosis was supported by demonstration of IgE-mediated sensitivity to the offending fruit (Indian jujube) through positive SPT to Indian jujube cocktail and Indian jujube itself. Other potential causes such an allergy to Thai and Indian sweetener was ruled out. Another limitation of the diagnostic work up was the lack of an oral challenge with Indian jujube which the patient declined.

Literature review demonstrated a few published reports of Indian jujube allergy. Lee et al. reported two cases of latex allergic patients who reacted to consumption of the fruit. One patient reacted with worsening of their atopic dermatitis and the other patient developed recurrent angioedema of the lips, tongue and throat [2]. Same authors described a case series of ten latex allergic patients who developed symptoms of upper (allergic rhinitis, oral allergy syndrome) and lower respiratory tract (asthma) after consumption of this fruit [4]. Similar to our case report, a case of systemic anaphylaxis to ingestion of five Indian jujubes in a 28-year-old female has been described. The patient went on to develop angioedema, generalized urticaria, chest tightness and hypotension a few minutes after the consumption of the fruit [3].

Unlike our patient, all cases reported thus far have occurred in patients who have a concomitant latex allergy [1–4]. Approximately 30–50% of patients who are allergic to latex have evidence of a coexisting food allergy, which is defined as latex-fruit syndrome [7]. This syndrome was first described in 1994 after a high number of patients with a fruit allergy were found to have a latex allergy. The common fruits identified include banana, avocado, chestnut, passion fruit, fig, pineapple, kiwi, potato, papaya, peach, grape, orange, tomato, melon, celery and peanut among others. *Ziz m 1* (30 kD), the major Indian jujube allergen identified, has been found to have sequence identity to many plant class III chitinases including latex *hevamine*. This protein possesses IgE binding capacity and inhibition studies have revealed evidence of cross-reactivity with the latex allergen [2–5]. Additionally, it has been proposed that a 20 kD *prohevein*-like protein may also be implicated in the cross-reactivity [3]. Our patient does not have a latex allergy, suggesting that there might be another unknown component of the Indian jujube allergen responsible for the systemic IgE-mediated reaction he experienced.

Two patterns of IgE sensitization have been recognized in food allergy. Primary sensitization is caused by ingestion of the specific food and secondary sensitization occurs as a result of cross-reacting aeroallergen antigens, mainly in the setting of oral allergy syndrome, not excluding anaphylaxis [8]. Interestingly, our patient grew up with a *Z. Mauritiana Lam* tree in his backyard and regularly consumed Indian jujubes throughout his life until he moved to Canada, where they were not readily available. We hypothesize that the cessation of regular consumption of Indian jujubes led to the development of primary sensitization to Indian jujube and subsequent anaphylaxis upon re-exposure.

To our knowledge, this is the first reported case of anaphylaxis to Indian jujube in a patient with no evidence of a co-existing latex allergy. Although a rare phenomenon, anaphylaxis secondary to ingestion of Indian jujube should be taken into consideration when working up patients presenting with anaphylaxis, especially those of Asian and Indian descent. Further studies are needed to help elucidate the underlying mechanism of this fruit’s ability to trigger systemic anaphylaxis.

## Data Availability

Not applicable.
